# 1723. Varicella in Uruguay. Epidemiological situation and prevention strategies. Period 2008 to 2021.

**DOI:** 10.1093/ofid/ofad500.1555

**Published:** 2023-11-27

**Authors:** Monica Pujadas Ferrer, Marcos Delfino, Federica Badia, Juan Kenny, Elizabeth Assandri, Ignacio Olivera, Catalina C Canziani, María Catalina Pírez

**Affiliations:** Faculty of Medicine University of the Republic Uruguay, Montevideo, Montevideo, Uruguay; Clínica Pediátrica, Facultad de Medicina, Universidad de la Repùblica, Montevideo, Montevideo, Uruguay; Facultad de Mediciina, Universidad de la Republica., Montevideo, Montevideo, Uruguay; Facultad Medicina, Universidad de la Repùblica, Uruguay, Montevideo, Montevideo, Uruguay; University of the Republic. Uruguay, Montevideo, Montevideo, Uruguay; Centro de Investigaciones Económicas (CINVE), Montevideo, Montevideo, Uruguay; Facultad de Medicina, Universidad de la Repùblica, Paysandú, Paysandu, Uruguay; Faculty of Medicin, University of the Republic, Montevideo, Montevideo, Uruguay

## Abstract

**Background:**

In Uruguay varicella is mandatory notification disease since 1963. Universal vaccination starts in 1999 (OKA strain 1 dose) when incidence was 105/100.000 inhab; in 2014 2^nd^ dose was added (5 years) to prevent outbreaks. Objective: describe epidemiology and variations of the cases notified in Uruguay between 1.1.2008 and 12.31.2021 according vaccine strain.

**Methods:**

Observational, descriptive, retrospective study. All cases reported to the Department of Health Surveillance (DEVISA)-Epidemiology Division-Ministry of Health in period: 1.1.08 to 12.31.21. Data source: DEVISA and Honorary Commission-Fight Against TB-Prevalent Diseases. Variables: notified cases, average annual incidence, age, sex, outbreaks. Vaccine: doses, OKA or MAV/06 strain Statistical analysis: frequency distribution, summary measures and statistical significance tests (significant: p value ≤ 0.05) 4 periods according vaccine strain in nation immunization program were defined: 2008 and 2009 (Oka), 2010 to 2012 (Oka and MAV06); 2013 to 2018 (MAV) and 2019 to 2021 (OKA)

**Results:**

Accumulated cases: 13,896 (annual average: 992). Women: 6639 (48%) Vaccine coverage was around 95-97%. Incidence: 32.2/100,000/inhabitants (95%CI:30-34) in 2008-2009, the rate decrease in 2010-2012 to 21.5 (19-13), in 2013-2018 increased 35,78 (35-36), in this period in 2013, 1968 cases was reported. Rate in 2019-2021: 12 (11-12) [2019: 24, 2020: 7 and 2021: 4,6], with 2 doses of vaccine. There wasn’t a significant difference in annual average cases (AANC) 2008-2013 (AANC: 1025) vs 2014-2018 (AANC: 1293) p=0,345. The AANC in 2019-2021 were 424. [rate in 2019: 24, in 2020: 7 and 2021: 4,6]. There was a significant increase in the annual average of outbreaks 30/2012-2013 vs. 90/2014-2018. In 2019:81, 2020:10 and 2021:7 outbreaks. Between 2008-2018 most cases were reported in the group 10 to 14 years of age (median 187.5m); 2019-2021, 678/1,272 cases were older than 12

**Figure:**
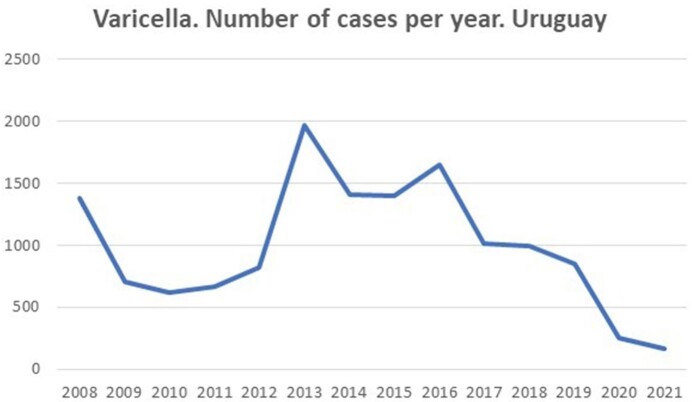

**Figure:**
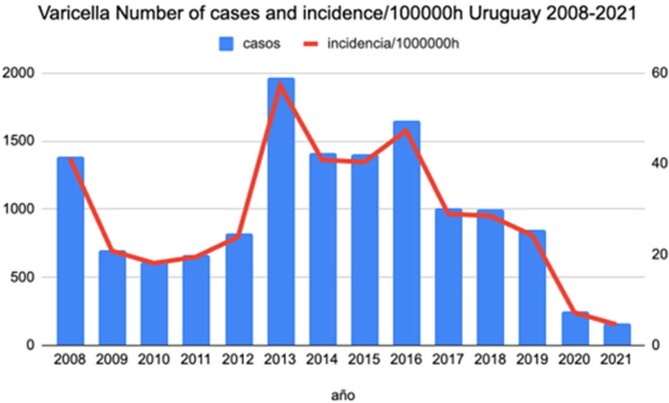

**Conclusion:**

Incidence dropped from 105 in 1999 to 24 in 2019. There was a displacement to older ages. The 2nd dose didn’t change incidence and outbreaks frequency. The incidence decreased in 2019-2021. Surely the great decrease in the incidence and outbreaks in 2020 and 2021 was influenced by COVID-19 pandemic. Since 2010 to 2018, the most widely used vaccine contained MAV/06

**Disclosures:**

**Marcos Delfino, Pediatrician, pediatric infectologist, Adjunct Professor of pediatric clinic**, Pfizer: Finnancial support to travel to IDWeek 2023 **Ignacio Olivera, Medical Doctor, Master in Health Economics and Pharmaceutical Economics**, Pfizer: Grant/Research Support|Roche: Expert Testimony|Sanofis Pasteur: Grant/Research Support **María Catalina Pírez, Pediatrician, Pediatric infectologist, microbiologist Professor of pediatric, Degree V**, Merck, Pfizer: Expert Testimony|Merck, Pfizer: Honoraria

